# Divide and conquer: processive transport enables multidrug transporters to tackle challenging drugs

**DOI:** 10.15698/mic2014.10.172

**Published:** 2014-09-23

**Authors:** Nir Fluman, Eitan Bibi

**Affiliations:** 1 Department of Biological Chemistry, Weizmann Institute of Science, Rehovot, Israel 76100.

**Keywords:** multidrug resistance, multidrug transport, MdfA, dequalinium, antibiotics, secondary transport, drug-proton exchange

## Abstract

Multidrug transporters are membrane proteins that catalyze efflux of antibiotics and other toxic compounds from cells, thereby conferring drug resistance on various organisms. Unlike most solute transporters that transport a single type of compound or similar analogues, multidrug transporters are extremely promiscuous. They transport a broad spectrum of dissimilar drugs and represent a serious obstacle to antimicrobial or anticancer chemotherapy. Many challenging aspects of multidrug transporters, which are unique, have been studied in detail, including their ability to interact with chemically unrelated drugs, and how they utilize energy to drive efflux of compounds that are not only structurally but electrically different. A new and surprising dimension of the promiscuous nature of multidrug transporters has been described recently: they can move long molecules through the membrane in a processive manner.

Except for their promiscuous nature, multidrug transporters probably work like any other transporter. Phylogenetically, they are members of large solute transporter families that comprise mostly substrate-specific members. For most transporters, including those that belong to the MFS superfamily of secondary transporters, a mechanism is suggested in which binding of substrate occurs on one side of the membrane, followed by transition into a substrate-occluded state and its release on the other side. Substrate binding and release are achieved by alternating access of binding sites to either side of the membrane by reciprocal opening and closing of the occluded conformer on the opposing sides of the membrane (Fig. 1A). In addition, many multidrug transporters can also bind hydrophobic drugs from within the membrane bilayer and export them to the medium. Secondary efflux in bacteria is often driven by the proton electrochemical gradient (∆µ̃_H_+, interior negative and/or alkaline). By this means, the free energy released by the inward downhill flux of protons with ∆µ̃_H_+ is utilized by the multidrug transporter to drive efflux of the drug (Fig. 1A). One critical feature of these so-called antiporters is that they never allow formation of a continuous channel across the membrane; the transport pathway is always blocked from either the intracellular or extracellular side of the membrane (Fig. 1A). This tight seal is crucial for preventing non-specific leakage of ions through the antiporter, which may collapse ∆µ̃_H_+. However, the architecture of these proteins restricts the size of the transported substrate: only those that can be accommodated structurally in the occluded state can be transported. Therefore, current wisdom suggests that processive transport of long substrate molecules in several cycles should be impossible.

**Figure 1:**
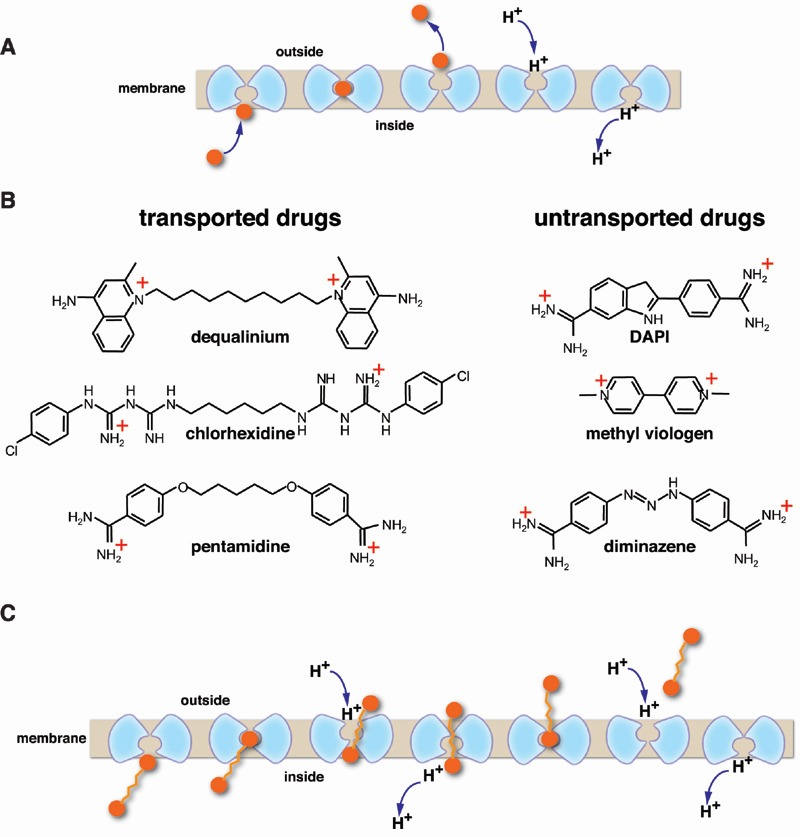
FIGURE 1: **(A) **General mechanism for H^+^-coupled solute antiport. Note that most transporters have an internal 2-fold symmetry axis and a monomeric transporter is represented here by two symmetrical parts, shown in light blue. The substrate is indicated by orange sphere. The occluded state is second from the left. **(B)** Chemical structure of divalent cationic drugs that are transported or untransported by MdfA. **(C)**Illustration of the suggested processive antiport mechanism.

The current study by Fluman *et al.* was sparked by the finding that MdfA, an *E. coli* multidrug antiporter, transports a divalent cationic drug that should not be transported. A number of studies indicate that MdfA couples the export of a single drug molecule with the import of a single H^+^. In principle, this coupling should prevent MdfA from catalyzing efflux of divalent cationic drugs, since these drugs would require simultaneous import of at least two H^+^ to maintain electroneutrality. Indeed, previous studies indicated that MdfA cannot transport such substrates. Therefore, the discovery that MdfA transports the divalent cationic drug dequalinium was surprising and suggested an un-orthodox mechanism. Upon further characterization, we found that dequalinium is not the only divalent cationic drug that is transported by MdfA. The transported divalent cationic drugs are from different pharmacological classes, but they share a striking chemical property: in each substrate, the two positively charged moieties are separated by a long, flexible carbon chain (Fig. 1B). This unique architecture is a vital clue for understanding the underlying transport mechanism.

Studies of the transport mechanism establish that unlike the usual MdfA substrates, which have a 1:1 drug:H^+^ stoichiometry, the divalent drugs exhibit a 1:2 stoichiometry. Since MdfA functions as a monomer, the finding suggests that divalent drugs are likely transported in a processive-like mechanism involving two consecutive transport cycles in which each cationic moiety is transported as if it were a separate substrate (Fig. 1C). Further studies lend strong support to this model by showing that other divalent substrates with a similar architecture are transported with a drug:H^+^ stoichiometry greater than unity not only by MdfA, but also by another multidrug antiporter. Collectively, the study indicates that multidrug antiporters are inherently capable of processivity.

As discussed above, it has been generally believed that processive solute transport is unlikely, since all transporters are tightly sealed either on the extracellular or intracellular side of the membrane. It now seems that at least narrow carbon chains that bridge positively charged moieties can be threaded through the pathway without compromising the seal (Fig. 1B,C). Such carbon chains are particularly suitable for this task, because they are narrow and may be accommodated inside the protein with relatively minimal structural perturbation. Another important aspect is the hydrophobic nature of the carbon chain, which may allow favorable hydrophobic interactions with the interior of the antiporter, as happens with lipids. Theoretically, the carbon chain of these substrates mimics the carbon chains of lipids, which are known structural, chemical, and even functional counterparts of integral membrane proteins, including solute transporters.

In summary: (i) Multidrug antiporters appear to have an even broader substrate-profile than previously recognized, and the architecture of the substrate may alter the stoichiometry of the transport mechanism. (ii) Antiporters are capable of processive transport, and they may even be able to transport much larger molecules or polymers, utilizing the same transport mechanism as they use for small solutes.

